# Glioblastoma disrupts the ependymal wall and extracellular matrix structures of the subventricular zone

**DOI:** 10.1186/s12987-022-00354-8

**Published:** 2022-07-11

**Authors:** Emily S. Norton, Lauren A. Whaley, María José Ulloa-Navas, Patricia García-Tárraga, Kayleah M. Meneses, Montserrat Lara-Velazquez, Natanael Zarco, Anna Carrano, Alfredo Quiñones-Hinojosa, José Manuel García-Verdugo, Hugo Guerrero-Cázares

**Affiliations:** 1 Department of Neurosurgery, Mayo Clinic Florida, 4500 San Pablo Road, Jacksonville, FL 32224 USA; 2 Neuroscience Graduate Program, Mayo Clinic Graduate School of Biomedical Sciences, Mayo Clinic, Jacksonville, FL USA; 3 Regenerative Sciences Training Program, Center for Regenerative Medicine, Mayo Clinic, Jacksonville, FL USA; 4 Department of Biology, University of North Florida, Jacksonville, FL USA; 5 Laboratory of Comparative Neurobiology, Cavanilles Institute of Biodiversity and Evolutionary Biology, University of Valencia, CIBERNED, Paterna, Spain; 6 Department of Neuroscience, Mayo Clinic, Jacksonville, FL USA; 7 Department of Cancer Biology, Mayo Clinic, Jacksonville, FL USA; 8 Biochemistry and Molecular Biology Graduate Program, Mayo Clinic Graduate School of Biomedical Sciences, Mayo Clinic, Jacksonville, FL USA

**Keywords:** Lateral ventricle, Stem cell niche, Subependymal zone, Glioma, Cerebrospinal fluid (CSF), Lipid droplets, Cilia

## Abstract

**Background:**

Glioblastoma (GBM) is the most aggressive and common type of primary brain tumor in adults. Tumor location plays a role in patient prognosis, with tumors proximal to the lateral ventricles (LVs) presenting with worse overall survival, increased expression of stem cell genes, and increased incidence of distal tumor recurrence. This may be due in part to interaction of GBM with factors of the subventricular zone (SVZ), including those contained within the cerebrospinal fluid (CSF). However, direct interaction of GBM tumors with CSF has not been proved and would be hindered in the presence of an intact ependymal cell layer.

**Methods:**

Here, we investigate the ependymal cell barrier and its derived extracellular matrix (ECM) fractones in the vicinity of a GBM tumor. Patient-derived GBM cells were orthotopically implanted into immunosuppressed athymic mice in locations distal and proximal to the LV. A PBS vehicle injection in the proximal location was included as a control. At four weeks post-xenograft, brain tissue was examined for alterations in ependymal cell health via immunohistochemistry, scanning electron microscopy, and transmission electron microscopy.

**Results:**

We identified local invading GBM cells within the LV wall and increased influx of CSF into the LV-proximal GBM tumor bulk compared to controls. In addition to the physical disruption of the ependymal cell barrier, we also identified increased signs of compromised ependymal cell health in LV-proximal tumor-bearing mice. These signs include increased accumulation of lipid droplets, decreased cilia length and number, and decreased expression of cell channel proteins. We additionally identified elevated numbers of small fractones in the SVZ within this group, suggesting increased indirect CSF-contained molecule signaling to tumor cells.

**Conclusions:**

Our data is the first to show that LV-proximal GBMs physically disrupt the ependymal cell barrier in animal models, resulting in disruptions in ependymal cell biology and increased CSF interaction with the tumor bulk. These findings point to ependymal cell health and CSF-contained molecules as potential axes for therapeutic targeting in the treatment of GBM.

**Supplementary Information:**

The online version contains supplementary material available at 10.1186/s12987-022-00354-8.

## Background

Glioblastoma (GBM) is the most common and lethal primary brain malignancy in adults [1]. Despite an aggressive treatment approach consisting of surgery, chemotherapy, and radiotherapy, the median survival remains just under 15 months, largely due to invasive brain tumor initiating cells (BTICs) escaping resection and leading to high rates of tumor recurrence [2, 3]. Intriguingly, the location of GBM tumors plays a pivotal part in patient prognosis. Tumors located in proximity to the lateral ventricles (LVs) result in increased expression of stem cell markers, increased distant recurrence, and decreased overall survival in GBM patients compared to LV-distal tumors [4–9]. Currently the reason for increased malignancy in these tumors has not been fully elucidated but may be due in part to interaction with factors of the subventricular zone (SVZ), including the cerebrospinal fluid (CSF) [10–13].

The SVZ is the largest neurogenic niche in mammals and contains a population of neural stem cells (NSCs) throughout life. These NSCs have been tied to GBM progression [14, 15] and have been identified as a likely cell-of-origin for these tumors in humans [16, 17]. The SVZ also contains a monolayer of multiciliated ependymal cells separating all but the thin apical processes of NSCs from the lumen of the LV [18, 19]. Ependymal cells are responsible for the movement of the CSF throughout the ventricular system, as well as establishing a selective interface that mediates bidirectional transport of ions, proteins, and fluid between the CSF and the brain parenchyma [20–22]. Loss of this ependymal cell layer results in dysfunctional CSF-interstitial fluid (ISF) exchange and impaired clearance of fluid and metabolites from the brain [23–27]. The disruption of this cell population has also been tied to increased oxidative stress and the accumulation of lipid droplets (LDs) [28], which has been subsequently connected with decreased neurogenic proliferation [29, 30].

Ependymal cells have another role in generating the specialized extracellular matrix (ECM) of the SVZ called fractones due to their fractal ultrastructure [31, 32]. These highly branched structures are able to bind and capture CSF-contained factors, such as FGF2 and BMP4, through high levels of fractone N-sulfated heparan sulfate proteoglycans (NS-HSPGs) interacting with cytokine heparin-binding domains [33–35]. The association of heparin-binding molecules with fractones profoundly affects SVZ niche homeostasis, particularly the proliferation of SVZ NSCs [33, 35]. Interestingly, alterations in fractone size and number have been reported in various conditions and disorders, including aging, autism, and hydrocephalus [36–38]. These structures have been theorized to contribute to GBM malignancy [12]; however, the role of fractones in LV-infiltrating GBM has not been explored.

We have recently shown that LV-proximal GBM disrupts the neurogenic cells of the SVZ in an immunocompromised rodent model [39]. Additionally, we have found that exposure to human CSF and CSF-contained factors increases the malignant behavior of patient-derived GBM cells [10, 11]. However, it is still unknown how GBM cells may access the CSF compartment and/or its contained components in vivo. We hypothesize that LV-proximal GBM disrupts the ependymal cell barrier and takes advantage of fractone structures to access cytokines and chemokines present in the CSF. Here we examine the integrity of the ependymal cell barrier and its produced extracellular matrix structures in the presence of nearby GBM.

## Materials and methods

### Cell culture

Patient-derived GBM BTIC line GBM1A, originally established as line 020913 [40] and extensively characterized by our collaborators, was cultured as neurospheres in Dulbecco's modified Eagle's medium/F-12 medium supplemented with EGF and FGF (20 ng/mL each). To localize cells in vivo, cells were transduced with a lentivirus for GFP-luciferase (GFP-luc; RediFect™ Red-FLuc-GFP, Perkin Elmer CLS960003) and sorted using fluorescence-activated cell sorting for GFP.

### Experimental animals

Animal experiments were approved by the Mayo Clinic Institutional Animal Care and Use Committee. Mice were housed in an AAALAC-accredited facility abiding by all federal and local regulations. Male immunosuppressed athymic nude mice (J:NU; Jackson Laboratory strain 007850) were maintained at Mayo Clinic Jacksonville with ad libitum access to food and water and a 12-h light–dark cycle. Animals were injected with GBM1A GFP-luc + BTICs for experiments at 6 weeks of age.

### BTIC xenograft and euthanasia

Mice were anesthetized with isoflurane inhalation and placed into a stereotactic frame. 3.5 × 10^5^ GBM1A GFP-luc + BTICs were injected in 2 μL of sterile PBS at a rate of 0.5 μL/minute. Animals were randomly assigned to one of three groups; LV-proximal vehicle injection (PBS), LV-distal GBM, and LV-proximal GBM (n = 20 per group total). LV-proximal and LV-distal surgical sites were established in the following coordinates in mm relative to bregma as previously described [39]; LV-proximal: AP: 1.0, L: 1.2, D: 2.3; LV-distal: AP: 1.0, L:2.1, D: 2.3. Mice were maintained for 4 weeks following tumor implantation for immunohistological analysis. Mice were then anesthetized with ketamine-xylazine and perfused with 0.9% saline followed by 4% paraformaldehyde (PFA). Brains were extracted and postfixed in 4% PFA overnight, then stored in PBS with 0.1% sodium azide at 4ºC. Animals used for wholemount experiments were perfused with 0.9% saline and brains were immediately processed as described below. Animals used for transmission electron microscopy analysis were perfused by 0.9% saline followed by 2% PFA/2.5% glutaraldehyde, then processed.

### Lateral ventricle wholemounts

The lateral wall of the LV was dissected out of the brain hemisphere ipsilateral to injection as described previously [41]. After dissection of the LV, wholemounts (n = 3 per group) were fixed overnight in 4% PFA. The next day, wholemounts were permeabilized by incubation in 0.1% Triton in PBS (PBS-TX), blocked for 1 h at room temperature in 10% normal donkey serum in PBS-TX, then incubated with primary antibodies at various concentrations (Table 1) for 3 days at 4 °C diluted in blocking solution. The wholemounts were then washed with PBS-TX and incubated with secondary antibodies in blocking solution at a concentration of 1:500 overnight at 4 °C protected from light. Wholemounts were then washed with PBS, counterstained with DAPI, and mounted on glass slides before imaging.

**Table 1:** List of primary antibodies used

Target	Species	Dilution factor	Catalog
Aquaporin-4 (AQP4)	Rabbit	1:1000	Atlas Antibodies #HPA014784
Beta-catenin (β-cat)	Rabbit	1:100	Cell Signaling Technology #9562
Connexin-43 (Cx-43)	Rabbit	1:1000	Sigma-Aldrich #C6219
Glial fibrillary acidic protein (GFAP)	Rat	1:250	Invitrogen #13-0300
Green fluorescent protein (GFP)	Rabbit	1:1000	Invitrogen #A11122
Laminin subunit gamma-1 (LMγ1)	Rat	1:250	Santa Cruz Biotechnology #sc-65643
N-sulfated heparan sulfate proteoglycans (NS-HSPGs)	Mouse	1:200	Amsbio #370255

List of antibody targets, host species, catalog number, and dilution factor used within the study

### Scanning electron microscopy

Animals (n = 3 per group) were perfused with 0.9% saline and tissue was fixed by immersion with 2% PFA/2.5% glutaraldehyde in 0.1 M phosphate buffer for 1 h. Samples were then post-fixed in 1% osmium tetroxide (Electron Microscopy Sciences) for 45 min at 4 °C. Samples were washed with deionized water and partially dehydrated in increasing concentrations of ethanol up to 100%. Subsequently, critical point drying and sputtering with gold/palladium alloy was performed at the Central Service for Experimental Research of the University of Valencia.

### Coronal immunohistochemistry (IHC)

Brains (n = 5 per group) were sectioned into 50 μm coronal sections using a Precisionary Compresstome VF-300-0Z vibrating microtome. Sections were permeabilized and blocked as described above. Sections were incubated with primary antibodies (Table 1) diluted in blocking solution overnight at 4 °C. The next day sections were washed and incubated with secondary antibodies (1:500 dilution) in the dark for 1 h at room temperature in blocking solution. Sections were then washed with PBS and counterstained with DAPI as a nuclear marker. For lipid droplet labeling, sections were permeabilized with 0.1% saponin and incubated with HCS LipidTOX Red Neutral Lipid Stain (Invitrogen H34476) diluted 1:100 in PBS for 1 h before mounting on glass slides.

### Fluorescent labeling of CSF-contacting cells with lipophilic dye

After 4 weeks of tumor growth, mice (n = 5 per group) were anesthetized and 1 µL of 0.2% 1,1'-Dioctadecyl-3,3,3',3'-Tetramethylindocarbocyanine (DiI) in 2% DMSO was injected into the contralateral ventricle from the original injection site using a stereotactic frame. Coordinates for the DiI injection site are the following mm from bregma: AP: − 0.5, L: − 0.7, D: 2.0. 24 h after injection, mice were perfused and brains were sectioned and processed for IHC.

### Transmission electron microscopy (TEM)

Samples (n = 3 per group) were sectioned into 200 µm sections and post-fixed with 2% osmium tetroxide (Electron Microscopy Sciences) for 2 h. Sections were then washed in deionized water, and partially dehydrated in 70% ethanol. Afterwards, the samples were contrasted with 2% uranyl acetate (Electron Microscopy Sciences) in 70% ethanol for 2 h at 4 °C. The samples were further dehydrated and infiltrated in Durcupan ACM epoxy resin (Sigma) at room temperature overnight, and then at 60 °C for 72 h. Once the resin was cured, SVZ sections were selected and cut into ultrathin Sects. (60–80 nm) using an Ultracut UC7 ultramicrotome (Leica Biosystems). These sections were placed on Formvar-coated single-slot copper grids (Electron Microscopy Sciences) and stained with lead citrate.

### Imaging and image processing

LV wholemount and coronal IHC preparations were visualized using a Zeiss LSM880 confocal microscope. For coronal sections, the entirety of the coronal SVZ was imaged dorsal to ventral on sections containing tumor or injection site. Images were taken using 10X, 20X, 40X, or 63X objectives. SEM images were obtained on a Hitachi S4800 microscope. TEM images were obtained with a FEI Tecnai Spirit G2 biotwin microscope with a Xarosa (20 Megapixel resolution) digital camera using Radius image acquisition software (EMSIS GmbH, Münster, Germany). ZEN Blue (Zeiss), ImageJ (NIH), and Vision4D (arivis) were used for image processing and quantification.

### Lipid droplet quantification

Images of lipid droplets along the entirety of the coronal SVZ section (dorsal to ventral) were acquired on the TEM. The number of lipid droplets per millimeter of SVZ was recorded. Images were opened with ImageJ. Scale was calibrated and lipid droplet area was measured using the oval selection tool.

### Fractone quantification

Images of fractones along the entirety of the coronal SVZ section (dorsal to ventral) were acquired using IHC and TEM. The IHC images were loaded into Vision4D software. The SVZ was manually selected and a fluorescence intensity threshold was set for laminin gamma-1 (LMγ1) and NS-HSPGs. Puncta expressing both markers above threshold were considered a single fractone. The number of fractones per millimeter of SVZ was recorded. TEM images were opened with ImageJ. Fractal structures with an electron dense layer surrounded by an electron lucent layer were defined as fractones [32]. Scale was calibrated and fractone area was measured using the freehand selection tool.

### Junction and channel quantification

Confocal images of ependymal cell junctions and channels were taken of the entirety of the ipsilateral SVZ section at the coronal section of injection at 20X magnification. The images were loaded into Vision4D software. The SVZ was manually selected and a fluorescence intensity threshold was set for each channel type. Puncta expressing markers above threshold were considered a single channel. Channel formations above the fluorescence threshold were automatically counted and recorded for analysis.

### Statistical analysis

All data is represented as the mean ± standard error of the mean unless otherwise indicated. Statistical analysis and graphical representation were performed using GraphPad Prism 9 ® software. Normal distribution of the data was evaluated using the Shapiro–Wilk normality test. For comparisons among three groups, analysis of variance (ANOVA) with Tukey’s post-hoc correction was performed. The level of significance was determined as p < 0.05.

### Data sharing

The data that support the findings of this study are available from the corresponding author upon reasonable request.

## Results

### The ependymal cells of the subventricular zone are physically disrupted by LV-proximal GBM

**Fig. 1:**
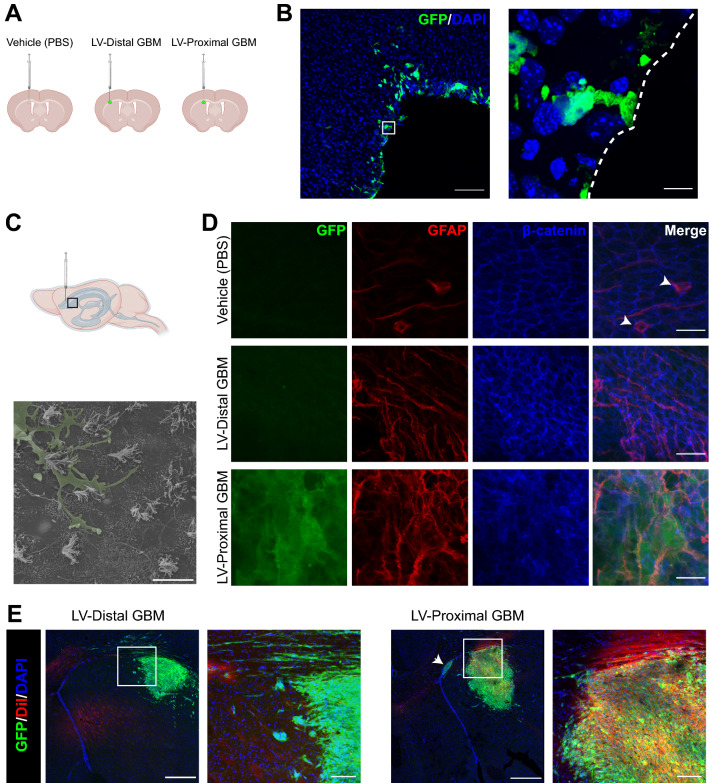
Ependymal cell lining of the lateral ventricle is disrupted by lateral ventricle-proximal glioblastoma. **A** Schematic of groups and injection sites. Created in Biorender.com **B** Representative coronal IHC image of multiple GFP + GBM cells in the parenchyma contacting the lateral ventricle in the LV-proximal group (n = 5). Scale bar = 100 µm. Zoomed in image shows a GFP + GBM cell extending a singular process to interact with the LV lumen. The lateral ventricle is designated with a a dashed white line. Scale bar = 10 µm. **C** Lateral ventricle cartoon displaying where images were taken for 1C-D. Scanning electron microscopy image of an invading cell on the ependymal cell apical surface (n = 3). Invading cell pseudocolored in green. Scale bar = 10 µm. **D** Representative lateral ventricle wholemount images in LV-proximal vehicle (top), LV-distal GBM (middle), and LV-proximal GBM (bottom) groups (n = 3). Pinwheel formations with GFAP + centers are indicated with white arrowheads. Scale bar = 20 µm. **E** Representative coronal immunohistochemistry images in LV-distal GBM (left) and LV-proximal GBM (right; n = 5 per group) showing increased DiI entry into LV-proximal GBM tumors and invading cells in zoomed out photo (arrow). Scale bar = 500 µm for zoomed out photos, 100 µm for zoomed in photos

We first evaluated the effect of tumor proximity to the LV on the integrity of the ependymal cell barrier of the SVZ. Patient-derived GBM BTICs modified to express GFP were implanted at locations proximal or distal to the LV (Additional file 1: Fig. S1). A vehicle injection of PBS at the LV-proximal injection site was also included to account for injection artifact (Fig. 1A). After 4 weeks, the LV lateral walls were extracted, and ependymal cell wall integrity was assessed through LV wholemount imaging. In the LV-proximal injection group, we identified single GFP + cells from LV-proximal tumors which extended single processes into the LV, interacting directly with the CSF in a manner reminiscent of NSCs (Additional file 4: Video S1). This result was corroborated through IHC on coronal sections, where single GFP + cells were again seen extending processes to the LV lumen and cells were seen invading into the LV (Fig. 1B). Additionally, using SEM we identified invading cells on the apical ependymal cell wall only in LV-proximal tumor animals, suggesting they belong to GBM cells (Fig. 1C). In the vehicle injection group, the ependymal wall remained intact as evaluated by immunofluorescent LV wholemount analysis. Ependymal cells were well-outlined with β-catenin and occasionally formed pinwheel formations with GFAP + centers (Fig. 1D top, Additional file 2: Fig. S2) and no GFP + cells are found within the tissue. In the LV-distal GBM group, the ependymal cell wall remained intact, but was accompanied by low levels of GFAP + astrocytic gliosis (Fig. 1D middle, Additional file 2: Fig. S2). In contrast, the LV-proximal tumor animals contained localized areas of extreme LV wall disruption, where ependymal cells were lost and GFP + GBM cells and several GFAP + astrocytes had direct contact with the LV lumen (Fig. 1D bottom, Additional file 2: Fig. S2). Interestingly, GFP + areas were located to the ipsilateral LV and were not observed in any of the surrounding ventricular spaces by IHC or the spinal canal by in vivo imaging signal, suggesting localized entry of GBM cells into the ipsilateral LV.

### LV-proximal GBM cells have direct contact with cerebrospinal fluid and its contained components

Given the disruption of the ependymal wall, we next assessed whether LV-proximal GBM cells therefore have direct access to CSF and CSF-contained components. Following 4 weeks of GBM growth, 1 µL of 0.2% DiI solution was injected into the contralateral LV from original injection and brains were analyzed for DiI fluorescence after 24 h. In the presence of LV-distal GBM, very few GFP + tumor cells were labeled by the DiI stain, and those labeled appeared to be migrating towards the LV (Fig. 1E, left). In contrast, LV-proximal GBM tumors occasionally had high numbers of LV-invading cells (Fig. 1E, right, arrow) and had increased numbers of GFP + tumor cells labeled with DiI (Fig. 1E, right), indicating direct contact of the tumors with CSF. This result proves that LV-proximal GBM tumors have access to the CSF compartment and strongly suggests that CSF-contained components are able to directly signal to LV-proximal GBM cells and vice versa.

### Nearby glioblastoma reduces the number and length of ependymal cell cilia

**Fig. 2:**
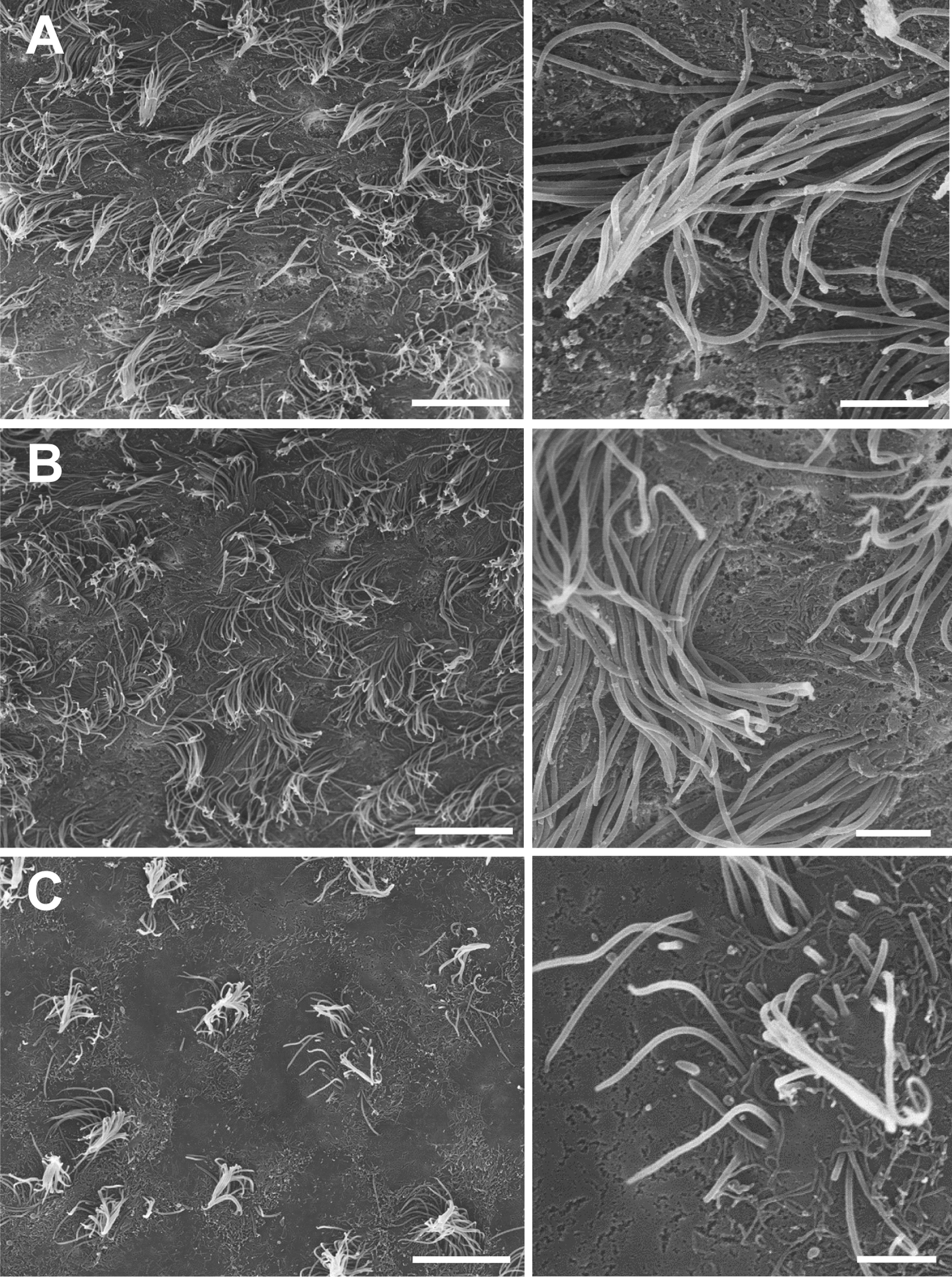
The ventricular wall presents cilia-devoid patches in LV-proximal GBM groups. Scanning electron microscopy images of the ependymal layer of mice injected with LV-proximal vehicle (**A**), LV-Distal GBM (**B**), and LV-Proximal GBM (**C**) (n = 3 per group). There is a progressive decrease in the number and uniformity of ependymal cilia that correlates with GBM-LV distance. Scale bars = 10 µm for larger image, 2 µm for zoomed photos. **A** Scanning electron microscopy images of LV-proximal vehicle injected mice showing an ependymal cell surface covered by healthy and directional cilia. **B** Scanning electron microscopy images of LV-distal GBM injected mice showing an ependymal cell surface covered by healthy cilia. **C** Scanning electron microscopy images of LV-proximal GBM injected mice showing an ependymal cell surface with unhealthy and short cilia. Scale bars = 10 µm for larger image, 2 µm for zoomed photos

After observing the physical disruption of the ependymal cell barrier and the accompanied invasion of GBM cells, we were interested to see if ependymal cilia would remain intact in the presence of LV-proximal GBM. SEM analysis of the ipsilateral LV wall was performed to examine the status of cilia. In the LV-proximal vehicle injected animals, cilia were abundant and long, with a uniform orientation (Fig. 2A). Cilia appeared similar in LV-distal GBM injected animals, with a slight loss of uniformity in the orientation of cilia (Fig. 2B). In the LV-proximal GBM injected animals, however, there was a stark reduction in the number of cilia (Fig. 2C). The cilia of this group also showed to be shorter and lack uniform directionality, suggesting alterations in CSF flow. Such a reduction in cilia, in both size and number, has clear implications for CSF regulation and flow throughout the ventricular system.

### Ependymal cells accumulate lipid droplets with increasing tumor proximity to the LV

**Fig. 3:**
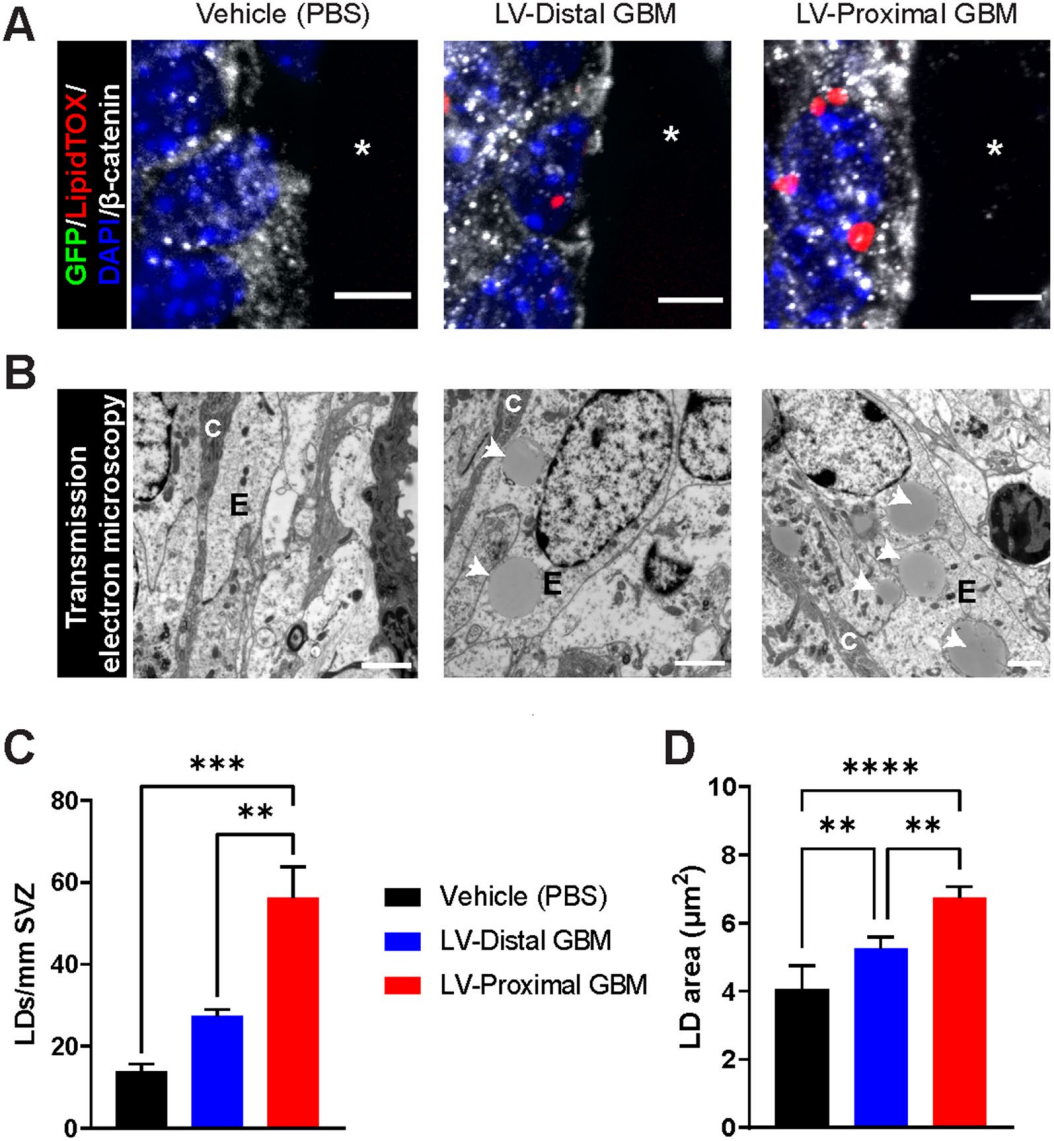
Lipid droplets accumulate in ependymal cells dependent on tumor proximity to the lateral ventricle. **A** Representative coronal immunohistochemistry images in vehicle (left), LV-distal GBM (middle), and LV-proximal GBM (right) showing the accumulation of lipid droplets within β-catenin + ependymal cells with increasing tumor proximity to the LV (n = 5). The lateral ventricle is indicated with a white asterisk. Scale bar = 5 µm. **B** Representative transmission electron microscopy images of the SVZ ependymal cells in vehicle (left), LV-distal GBM (middle), and LV-proximal GBM (right) showing lipid droplet accumulation with LV-proximal GBM (n = 3). Ependymal cells are indicated with “E”, and cilia are indicated with “C”. White arrows indicate lipid droplets. Scale bar = 2 µm. **C** Quantification of number of lipid droplets per mm of SVZ between vehicle, LV-distal GBM, and LV-proximal GBM groups measured using TEM analysis (n = 3 per group). **D** Quantification of lipid droplet area (µm^2^) between vehicle, LV-distal GBM, and LV-proximal GBM groups measured using TEM analysis (n = 3 per group). The data are presented as mean ± standard error of the mean. Statistical test used was ANOVA with Tukey’s post-hoc correction. **p < 0.01, ***p < 0.001, ****p < 0.0001. Non-significant interactions are not indicated

In previous works, reduction in cilia density has been tied to accumulation of LDs [42]. Therefore, we were next interested to investigate whether ependymal cells accumulated LDs in response to nearby GBM. To do so, we measured the incorporation of LDs in ependymal cells using both IHC and TEM. Using LipidTOX to fluorescently label neutral lipids in β-catenin-outlined ependymal cells, we identified an accumulation of ependymal LDs in LV-proximal GBM compared to vehicle-injected and LV-distal GBM groups (Fig. 3A). This was verified using TEM, where ciliated ependymal cells of the SVZ were shown to significantly accumulate round LDs of homogenous density in the LV-proximal GBM group compared to LV-distal GBM and vehicle-injected mice (mean ± SEM Vehicle: 13.95 ± 1.69 LDs/mm SVZ, LV-distal GBM: 27.34 ± 1.61 LDs/mm SVZ, LV-proximal GBM: 56.19 ± 7.67 LDs/mm SVZ, p < 0.01; Fig. 3B, C). Additionally, the average LD area was significantly increased both by the presence of GBM and increasing tumor proximity to the LV (mean ± SEM Vehicle: 4.062 ± 0.696 µm^2^, LV-distal GBM: 5.266 ± 0.324 µm^2^, LV-proximal GBM: 6.743 ± 0.328 µm^2^, p < 0.01; Fig. 3D). The accumulation of both number and size of LDs in the SVZ of LV-proximal GBM mice indicates increased stress in these cells, potentially due to physical disruption of the LV wall by tumor cells.

### Fractone extracellular matrix structures are altered by LV-proximal GBM

**Fig. 4:**
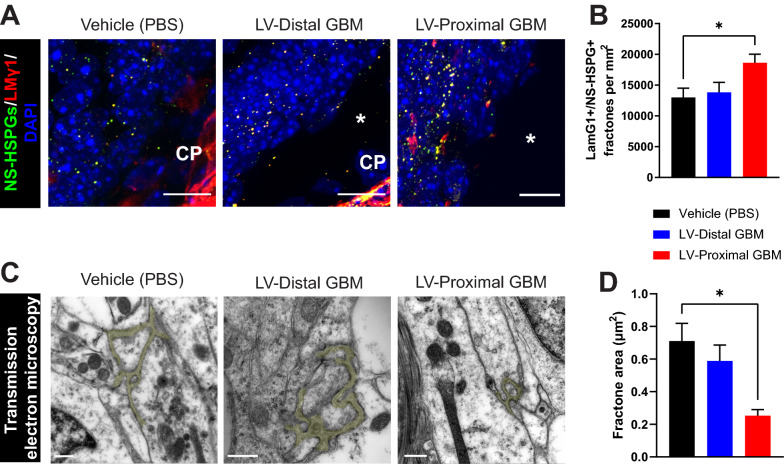
Fractone density and size are altered by LV-proximal GBM. **A** Representative coronal immunohistochemistry images in vehicle, LV-distal GBM, and LV-proximal GBM showing the density of fractone ECM structures in the SVZ increasing in LV-proximal GBM (n = 5). The lateral ventricle is indicated with a white asterisk. The choroid plexus is indicated as “CP”. Scale bar = 10 µm. **B** Quantification of the number of LMγ1 + /NS-HSPG + fractone structures. **C** Representative TEM images of fractones pseudocolored in yellow in vehicle (left), LV-distal GBM (middle), and LV-proximal (right) groups displaying decreased fractone size in LV-proximal GBM (n = 3). Scale bar = 2 μm. **D** Quantification of fractone area (µm^2^) measured using TEM. The data are presented as mean ± standard error of the mean. Statistical test used was ANOVA with Tukey’s post-hoc correction. *p < 0.05. Non-significant interactions are not indicated

Fractones, ECM structures derived from ependymal cells, have been previously shown to mediate signals from the LV to NSCs and have been hypothesized to play a role in GBM biology [12, 31, 33]. To determine whether the ependymal-derived fractone structures are disrupted in response to tumor proximity to the LV, we evaluated the number of LMγ1 + /NS-HSPG + puncta in the ependymal wall of the SVZ using IHC. Interestingly, we identified a significant increase in SVZ fractone density in LV-proximal GBM mice compared to vehicle and LV-distal GBM-injected mice (mean ± SEM Vehicle: 12,993 ± 1514 fractones/mm^2^ SVZ, LV-distal GBM: 13,826 ± 1620 fractones/mm^2^ SVZ, LV-proximal GBM: 18,644 ± 1388 fractones/mm^2^ SVZ, p < 0.05; Fig. 4A, B). Given the increase in fractone density, we were also interested in seeing if the size of these structures was changed in response to nearby GBM. Conversely, we found by TEM analysis that the average area of fractone structures is decreased in LV-proximal GBM compared to vehicle injected mice (mean ± SEM Vehicle: 0.710 ± 0.109 µm^2^, LV-distal GBM: 0.589 ± 0.097 µm^2^, LV-proximal GBM: 0.253 ± 0.037 µm^2^, p < 0.05; Fig. 4C, D).

We were also interested to see whether fractone localization is affected by the presence of a LV-proximal GBM tumor. Using TEM, we determined that in the vehicle and LV-distal GBM injected animals, fractones were localized to the base of ependymal cells as has been previously reported. In contrast, animals with LV-proximal GBM occasionally had displaced fractone localization, with fractones being directly adjacent to the LV (Additional file 3: Fig. S3). These data show that fractones may have increased access to CSF in the presence of LV-proximal GBM.

### Ependymal cell junctions and channels are compromised by lateral ventricle-proximal GBM

**Fig. 5:**
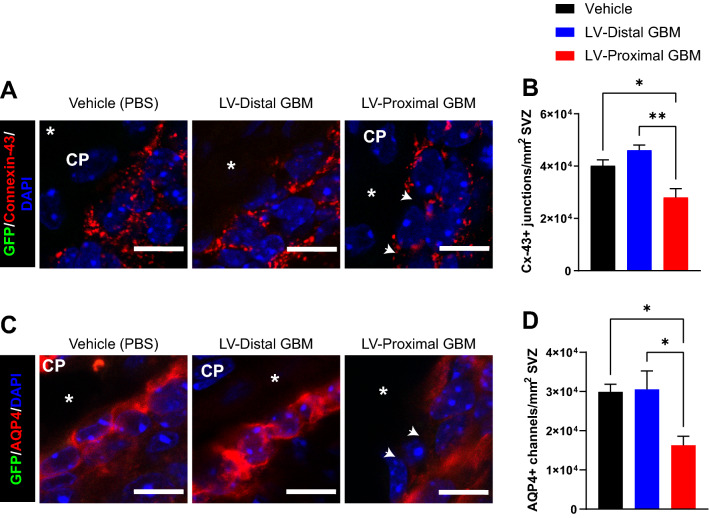
Ependymal cell channels are reduced with LV-proximal GBM. **A** Representative coronal immunohistochemistry images in vehicle (left), LV-distal GBM (middle), and LV-proximal GBM (right) groups of ependymal cell connexin-43 junctions showing loss of junctions in LV-proximal GBM (n = 5). White arrows indicate junctional loss. The lateral ventricle is indicated with a white asterisk. The choroid plexus is indicated as “CP”. Scale bar = 10 µm. **B** Quantification of number of Cx-43 + puncta. **C** Representative coronal immunohistochemistry images in vehicle (left), LV-distal GBM (middle), and LV-proximal GBM (right) groups of ependymal cell AQP4 channels showing loss of channels in LV-proximal GBM (n = 5). White arrows indicate channel loss. The lateral ventricle is indicated with a white asterisk. Scale bar = 10 µm. **D** Quantification of number of AQP4 + channels. The data are presented as mean ± standard error of the mean. Statistical test used was ANOVA with Tukey’s post-hoc correction. *p < 0.05, **p < 0.01. Non-significant interactions are not indicated

Disruptions in cell junctions between ependymal cells may contribute to compromised ependymal cell function and barrier integrity. In particular, functional Cx-43 junctions are important for reactivation of proliferation in the subependymal niche in response to injury [43, 44]. Given previous findings implicating decreased NSC proliferation in response to GBM [39, 45], we were interested in how these junctions may be altered by GBM proximity to the niche. To examine cell junction integrity in ependymal cells, we performed IHC for Cx-43 junctions in the SVZ. We identified a significant decrease in Cx-43 + junctions in the presence of LV-proximal GBM compared to other groups (mean ± SEM Vehicle: 40,132 ± 2287 Cx-43 + junctions/mm^2^ SVZ, LV-distal GBM: 46,053 ± 2029 Cx-43 + junctions/mm^2^ SVZ, LV-proximal GBM: 28,036 ± 3347 Cx-43 + junctions/mm^2^ SVZ, p < 0.05; Fig. 5A, B). The notable decrease in these junctions may partially drive the decreased neurogenesis in the SVZ previously observed in response to LV-proximal GBM.

Next, we were interested to examine other important ependymal cell channels, including AQP4 channels. These channels are integral for maintaining the structural and functional integrity of ependymal cells [46] and appear to co-regulate Cx-43 + gap junctions. IHC against AQP4 revealed a significant decrease in these channels in the LV-proximal GBM group compared to LV-distal GBM and LV-proximal vehicle (mean ± SEM Vehicle: 29,912 ± 1925 AQP4 + junctions/mm^2^ SVZ, LV-distal GBM: 30,561 ± 4682 junctions/mm^2^ SVZ, LV-proximal GBM: 16,278 ± 2318 junctions/mm^2^ SVZ, p < 0.05; Fig. 5C, D). The combined decrease of AQP4 channels and Cx-43 junctions suggests loss of ependymal cell barrier integrity with implications for CSF production and fluid exchange with the parenchyma.

## Discussion

LV-associated GBM is of great interest due to the increased malignancy of these tumors, resulting in worse outcome for patients. Tumor access to components of the SVZ, including the CSF, may partially explain increased malignancy and could result in potential therapeutic targets. In this study, we highlight the disruption of the ependymal cell monolayer of the SVZ in the presence of nearby GBM. Our results indicate that primary patient-derived GBM cells induce physical disruption of the ependymal cell wall, resulting in decreased functional cilia, an accumulation of lipid droplets and fractone structures, and an entry of CSF to the tumor mass. Together, these data point to ependymal cell health as a potential modulator of GBM malignancy.

Our data indicates that LV-proximal tumors physically disrupt the ependymal cell barrier, resulting in local areas of GBM cell contact with the LV lumen. GBM cells extend processes to interact with the CSF and, occasionally, invade to occupy the LV ependymal cell surface. This is in opposition to previously published work, where researchers have found that the ependymal cell monolayer actively prevents GBM penetration into the ventricle [47]. Based on our findings, we propose that there are only small regional areas of ependymal cell disruption where invading cells are able to penetrate into the ventricular lumen, thereby affecting the biology of many nearby ependymal cells. The effect on ependymal cell health from these few invading cells then contributes to CSF entry into the parenchyma and GBM progression. Despite our evidence that invading GBM cells enter the LV lumen, tumor invasion through the CSF into other parts of the brain and spinal cord is exceedingly rare in patients and animal models. Many have found that GBM cells are chemoattracted to components of the SVZ, including NSCs and CSF [10, 11, 48, 49]. Therefore, these invading tumor cells may remain chemoattracted to these components of the SVZ, thereby remaining in the SVZ and LV surface. Additionally, it is possible the lumen-contained GBM cells very rarely survive detaching from the LV wall or migrate long distances on the apical side of ependymal cells, preventing tumor expansion to other areas of the CNS.

The disruptions we observed in the ependymal cell layer strongly suggest the interaction of LV-proximal GBM with CSF. Using DiI labeling, we identified increased CSF entry into LV-proximal GBM compared to LV-distal GBM over a period of 24 h. CSF biology has been shown to contribute to GBM outcomes in patients; increased CSF volume in GBM patients is associated with decreased overall survival [50]. Additionally, previous studies have identified decreases in CSF outflow and turnover in murine models of GBM [51], but have not studied this in the context of tumor proximity to the LV. We have found that human CSF increases malignancy-promoting transcriptomic pathways in patient-derived GBM cells, and that the molecules regulated by CSF contribute substantially to cancer cell biology and patient outcomes [10, 11]. The findings in this study tying increased CSF interaction with LV-proximal tumors suggests that tumors in contact with the LV may have a specific transcriptomic signature contributing to malignancy that could be targeted by future therapeutics.

We have identified that LV-proximal GBM starkly decreases the number and length of ependymal cell cilia compared to the vehicle and LV-distal tumor injection groups. In the LVs, ependymal cells play a major role in circulating CSF through the coordinated beating of their many cilia. The number, length, and polarity of these ependymal cilia is important for the force and direction of CSF flow in brain homeostasis [52–55]. Additionally, the beating of ependymal cilia is required for the proper directional migration of neuroblasts down the rostral migratory stream towards the olfactory bulb during neurogenesis [22]. Due to our present findings and previous findings implicating LV-proximal GBM in decreased neurogenesis and oligodendrocyte differentiation [39], we propose that the patches of cilia-devoid ependymal cells in the presence of nearby tumors contribute to a change in CSF flow, thereby affecting downstream neurogenic processes. Altered neurogenesis, oligodendrocyte differentiation, and CSF flow would significantly alter brain homeostasis, potentially resulting in promoted tumor progression and increased clinical impact on patients.

We also identified changes in ependymal cell LDs depending on tumor proximity to the LV. As LV-GBM distance decreased, an increase in LDs number and size was observed. Accumulation of LDs has been tied to metabolic stress of ependymal cells as well as decreased proliferation of NSCs in the SVZ [28–30]. Although the mechanism tying high numbers of LDs to decreased neurogenesis is not fully elucidated, infusion of additional lipids into the LV results in decreased NSC proliferation and differentiation in cognitively normal mice via oleic acid-induced hyperactivation of AKT signaling [29]. This would indicate that NSCs rely on normal ependymal cell LD activity to function normally, and that the increase in LDs may be either tied to the decreased neurogenesis we have previously observed in this model [39]. However, due to the similarity between NSCs and GBM cells, it is interesting that GBM cells have not been found to reduce their proliferation in response to increased lipids. This may be due to a metabolic reliance of GBM cells on lipids for tumor progression [56–58], which may now be indicated as a potential therapeutic target for LV-associated GBM.

Our data also indicate an increase in the number of fractone ECM structures in the SVZ, but with a decrease in the measured size of these structures by TEM. Interestingly, our findings on alterations in extracellular matrix fractone structures contrasts those found in other pathologies involving the LV, such as aging and hydrocephalus [36, 38, 42]. In aging, for example, fractone number significantly decreases, but size dramatically increases and morphology of fractones is altered [34]. These changes in fractone number and structure are associated with decreased neurogenesis in the SVZ. Although we find the opposite fractone changes in our model, our previous findings and others support decreased SVZ neurogenesis in the presence of GBM. This may be due to a different cellular source of fractones in tumor pathology. GBM cells are able to secrete their own ECM, including components of laminin, fibronectin, and hyaluronic acid [59]. It is possible that local GBM cells infiltrating the SVZ are also able to secrete fractone-like structures which cannot be differentiated from those generated by ependymal cells [31], contributing to the higher fractone number. It is also possible that nearby GBM cells break up fractones produced by ependymal cells via secreted MMPs or other ECM-targeting enzymes, which could contribute to increased migration when in proximity to ECM components [60]. Additionally, GBM cells may hijack some of the communication fractones have with NSCs in the SVZ. GBM cells strongly proliferate in the presence of heparin-binding growth factors such as FGF2 that are captured by fractones in the normal brain [36]. Due to our findings that ependymal cell health is decreased and there are GBM cells in direct contact with the CSF, it may also be possible that heparin-binding factors contained within the CSF are bound to the increased number of fractones and are able to contribute to GBM malignancy. The biological mechanisms contributing to fractone alterations in the presence of tumors and how they contribute to GBM biology require further study in future works.

We have also identified a significant decrease in both Cx-43 + junctions and AQP4 + channels in LV-proximal GBM animals compared to LV-distal GBM and LV-proximal vehicle. These two membrane-contained proteins are co-regulated in ependymal cells, though the mechanism of this regulation is not fully described [46, 61]. Ependymal Cx-43 junctions have also been implicated in reactivating the proliferation of the neurogenic niche in response to injury or disease, where blocking Cx-43 signaling with hemichannel specific blockers prevents proliferation in response to spinal cord injury [43, 44]. The loss of these junctions likely contributes to the decreased proliferation in the SVZ we and others have found in previous work [39, 45]. Additionally, the ependymal AQP4 channels are closely tied to the structural and functional integrity of the ependymal cell monolayer, as well as proper CSF-ISF balance in the brain [46, 61, 62]. Genetic knockout of AQP4 results in disorganization of the ependymal cell layer [46], as well as significant alterations in the CSF production and absorption process [62]. The decrease in these proteins due to nearby GBM may contribute to CSF dysregulation that is seen in tumor patients [63], as well as to disorganization of the ependymal cell barrier.

Interestingly, several of our observations in LV-proximal GBM have ties to ependymal cells in aging. In normal and pathological aging, ependymal cells also accumulate lipid droplets that have associations with decreased neurogenesis [29, 42, 64]. Additionally, aged ependymal cells also have sparse, shorter cilia compared to young counterparts [42], similar to what we see in LV-proximal GBM. Other aspects of our work, such as decreased ependymal cell AQP4 junctions and an increase in small fractone structures, is opposite to what is seen in ependymal cell aging [36, 42, 65]. This indicates that though there are clear similarities between the effects of aging and LV-proximal GBM on ependymal cell health, the conditions are independent. Elucidating the mechanisms driving these changes in ependymal cell health in aging and GBM should be a priority of future work.

Although this study provides valuable and novel information on how decreased ependymal cell health may contribute to the formation of a GBM-CSF communication axis, this study has limitations in the use of an immunosuppressed animal model. The SVZ has a unique immune microenvironment [66] which may affect the communication between this area of the brain and an LV-proximal GBM. Additionally, the immune microenvironment plays a large role in GBM progression [67], and many of the transcriptional signatures in GBM regulated by CSF contact are related to inflammation [10]. Future studies would benefit from the inclusion of immunocompetent models. Additionally, this study lacks validation using human specimens due to the difficulty in obtaining histology-grade samples of the ependymal wall in GBM patients. There are important differences between the human and rodent SVZ. Although both have the lumen of the lateral ventricle lined by a monolayer of ciliated ependymal cells and contain a population of NSCs throughout life [68, 69], the NSCs of the human SVZ are largely quiescent, and humans lack a prominent and active rostral migratory stream throughout adulthood [70–72]. Importantly, the human SVZ contains a hypocellular gap directly beneath the ependymal layer, then followed by an astrocytic ribbon containing the NSCs [72, 73]. It is still unclear how the additional presence of the hypocellular gap and astrocytic ribbon would affect the invasion of GBM cells or the health of the ependymal cell layer in the presence of LV-proximal GBM. Further research using human specimens is required to determine whether decreased ependymal cell health drives GBM-CSF interaction in patients.

## Conclusions

**Fig. 6:**
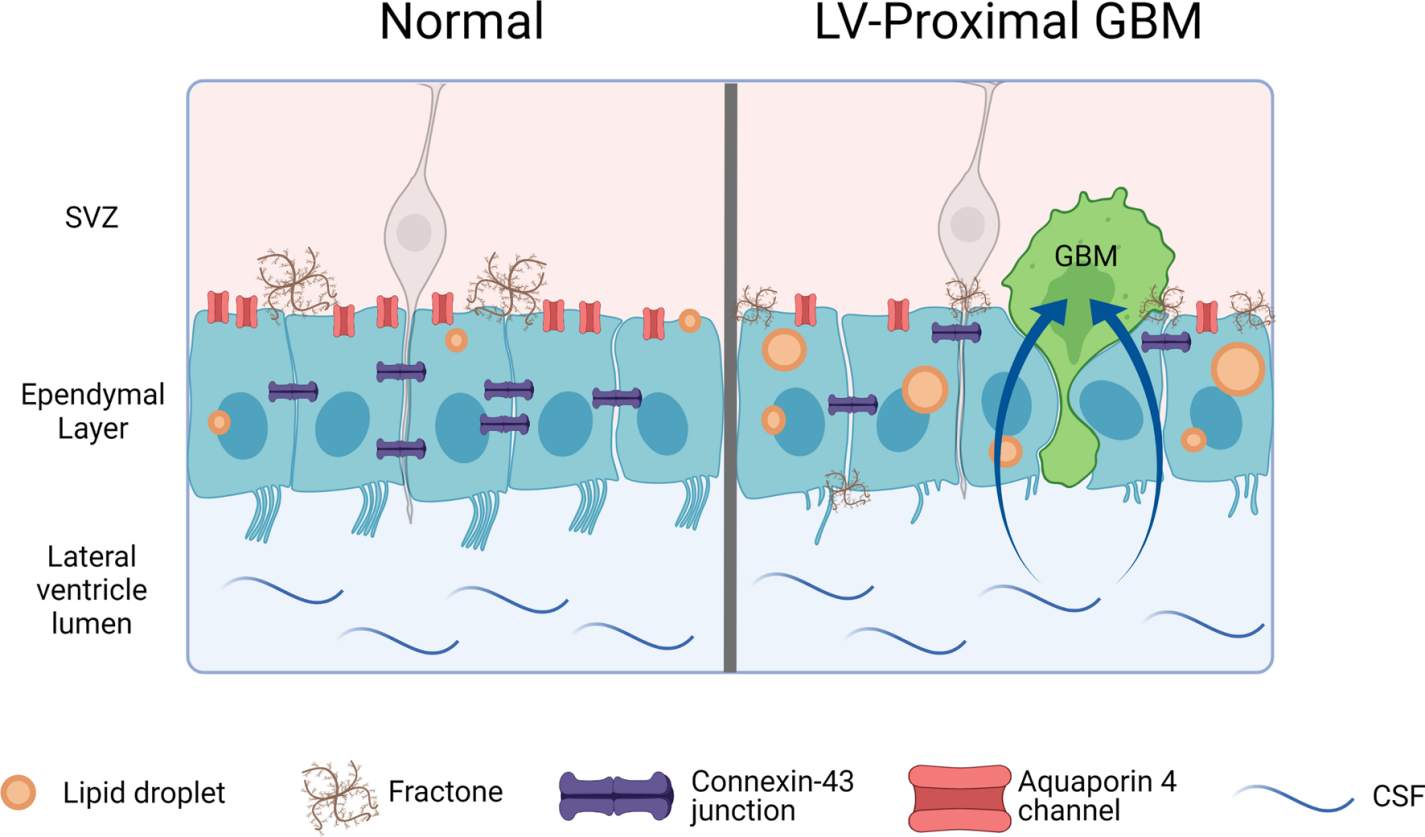
Graphical summary. Schematic diagrams of the normal ependymal cell barrier in the SVZ (left) and the disrupted ependymal cell barrier and accompanying extracellular matrix in the presence of a LV-proximal GBM (right). Created with BioRender.com

Ultimately, our results indicate that GBM cells are able to disrupt the LV wall by damaging ependymal cells and their associated ECM structures. The disruption of the ependymal wall integrity results in the invasion of GBM cells to the LV lumen and infiltration of CSF into the tumor bulk (Fig. 6). The direct interaction of CSF and its contents and GBM cells may contribute to the increased malignancy of tumors observed in patients with LV-proximal GBM. Ultimately, a potential therapeutic approach in brain tumor patients may include targeting the GBM-CSF interaction with the use of specific inhibitors.

## Supplementary Information

**Additional file 1: Figure S1.** Representative photos of LV-Proximal and LV-Distal GBM at endpoint. Representative IHC images of LV-proximal and LV-Distal GBM tumors at endpoint to display heterogeneity of size and location to the LV. LV outlined with white dashed line. White “M” indicating medial wall of LV and white “L” indicating lateral wall of LV. Scale bar = 100 μm.

**Additional file 2: Figure S2.** Orthogonal views of LV wholemounts. Orthogonal images of the LV wholemounts in Fig. 1 to display the localization of astrocytes and GBM cells on the LV wall surface. Scale bar = 20 μm.

**Additional file 3: Figure S3.** Fractones are occasionally displaced to the LV wall in the presence of LV-proximal GBM. Representative TEM image of a fractone pseudocolored in yellow in the LV-proximal GBM group displaying a fractone localized to the LV wall (outlined in red, labeled LV). Scale bar = 2 μm.

**Additional file 4: Video S1.** GBM cells extend processes to contact the lateral ventricle from the brain parenchyma. GFP + GBM cells (in green) extend individual processes to the LV lumen, as indicated by reaching through the β-cat + ependymal cell layer (in blue).

## Data Availability

The datasets used and/or analyzed during the current study are available from the corresponding author on reasonable request.

## Abbreviations

BTIC: Brain tumor initiating cell
CSF: Cerebrospinal fluid
DiI: 1,1'-Dioctadecyl-3,3,3',3'-tetramethylindocarbocyanine
ECM: Extracellular matrix
GBM: Glioblastoma
IHC: Immunohistochemistry
ISF: Interstitial fluid
LD: Lipid droplet
LV: Lateral ventricle
NSC: Neural stem cell
PFA: Paraformaldehyde
SEM: Scanning electron microscopy
SVZ: Subventricular zone
TEM: Transmission electron microscopy
