# Supplementary figures and images for: Glioblastoma disrupts the ependymal wall and extracellular matrix structures of the subventricular zone
Source: Fluids Barriers CNS. 2022 Jul 11;19:58. doi: 10.1186/s12987-022-00354-8 (PMC9277938; doi:10.1186/s12987-022-00354-8)

LV-Proximal  
GBM

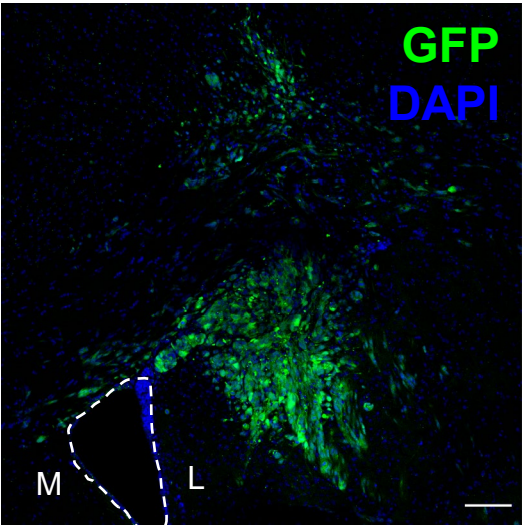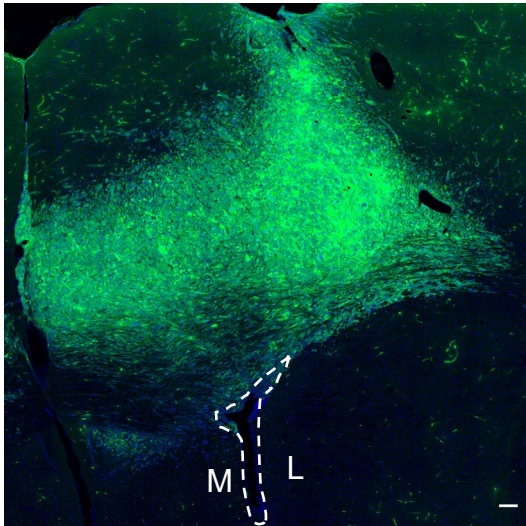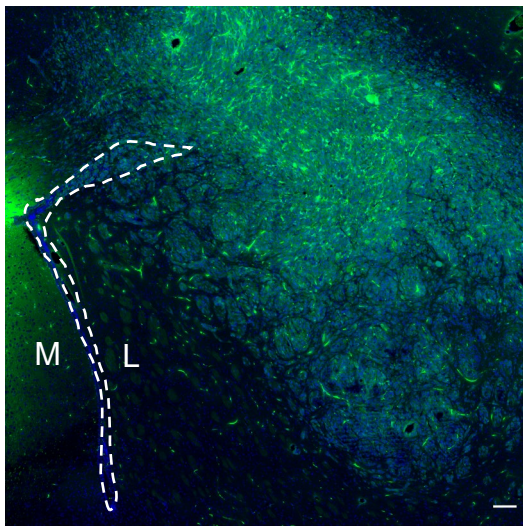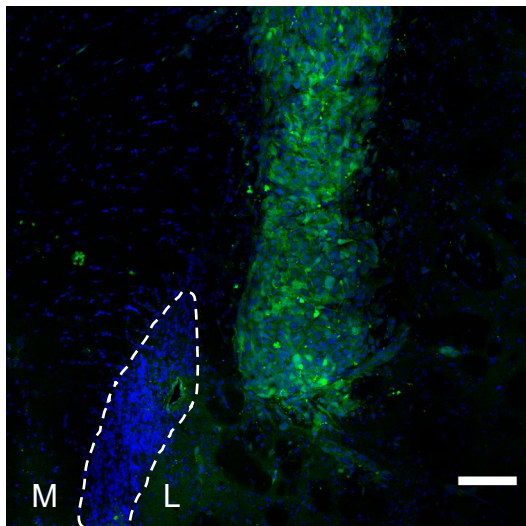

LV-Distal  
GBM

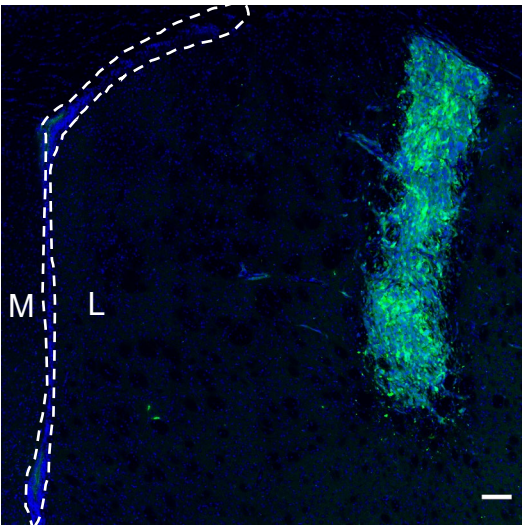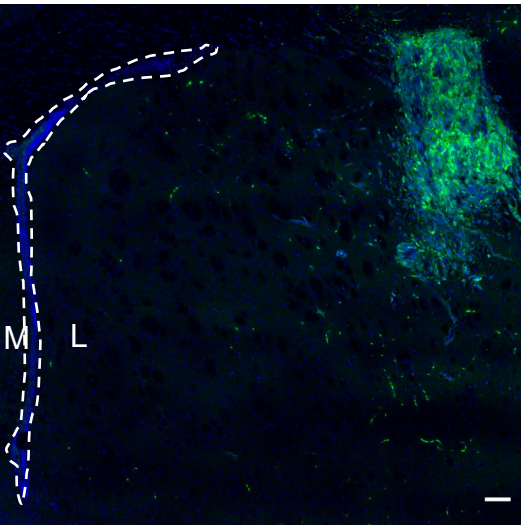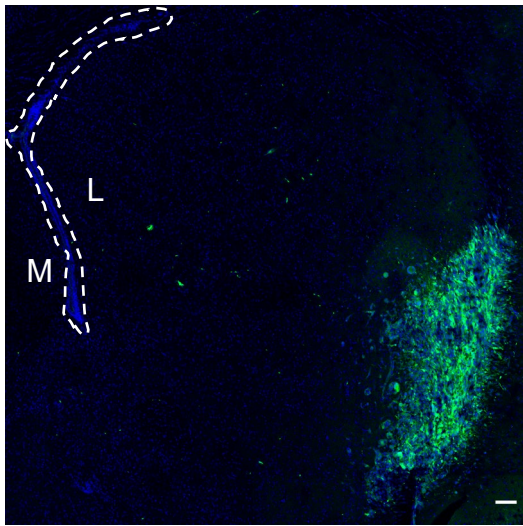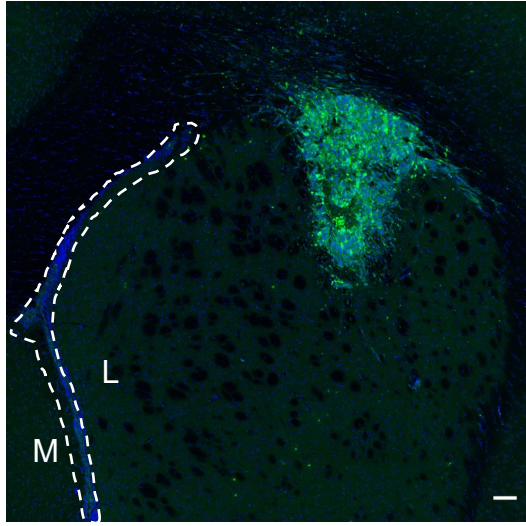

Supplement: Supplementary file 1 — Additional file 1: Figure S1. Representative photos of LV-Proximal and LV-Distal GBM at endpoint. Representative IHC images of LV-proximal and LV-Distal GBM tumors at endpoint to display heterogeneity of size and location to the LV. LV outlined with white dashed line. White “M” indicating medial wall of LV and white “L” indicating lateral wall of LV. Scale bar = 100 μm. [file 12987_2022_354_MOESM1_ESM.pdf]

Vehicle (PBS)

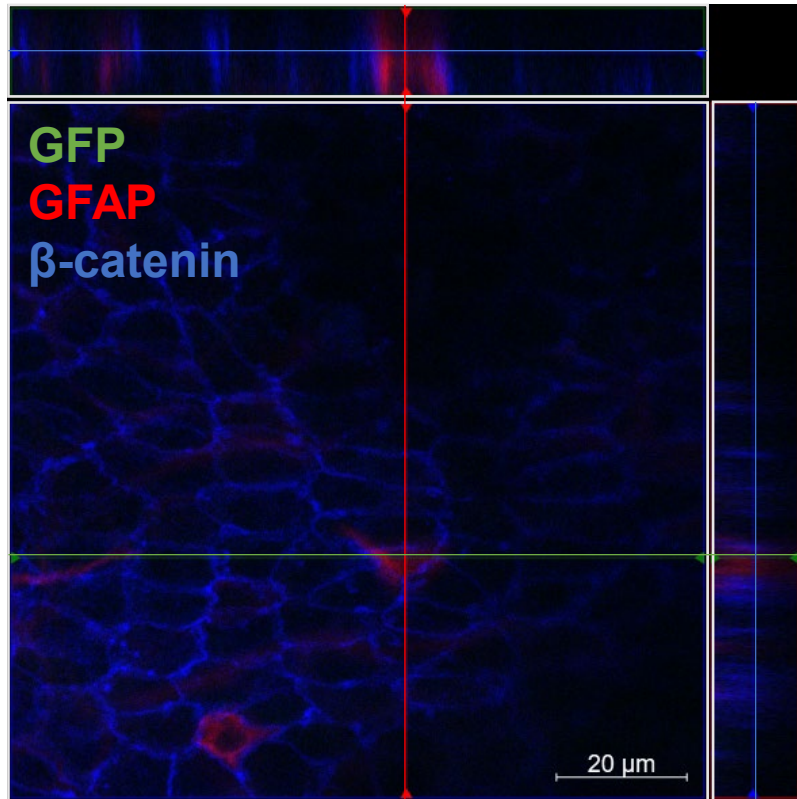

LV-Distal GBM

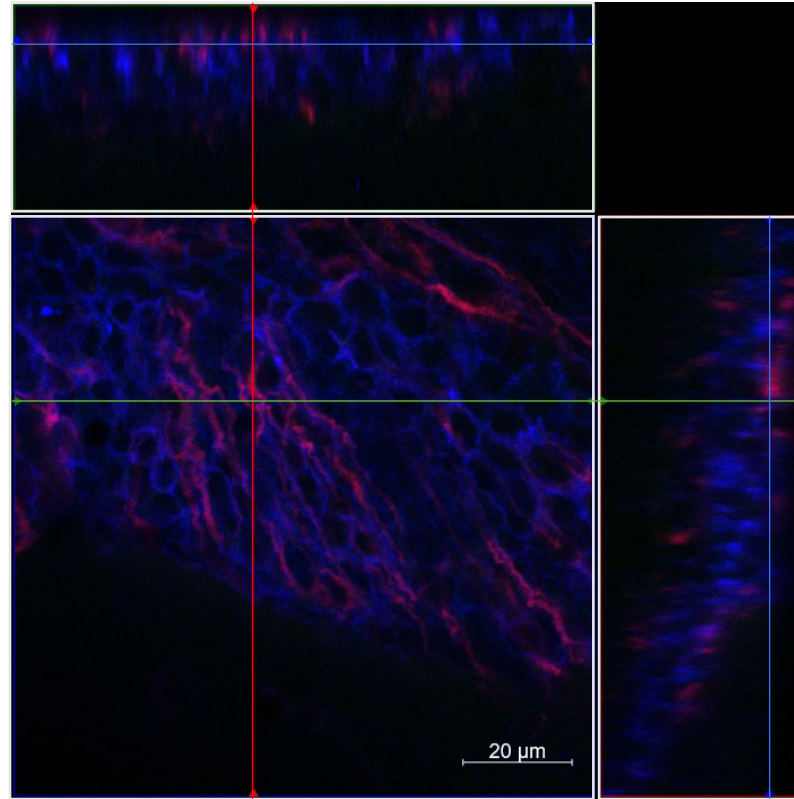

LV-Proximal GBM

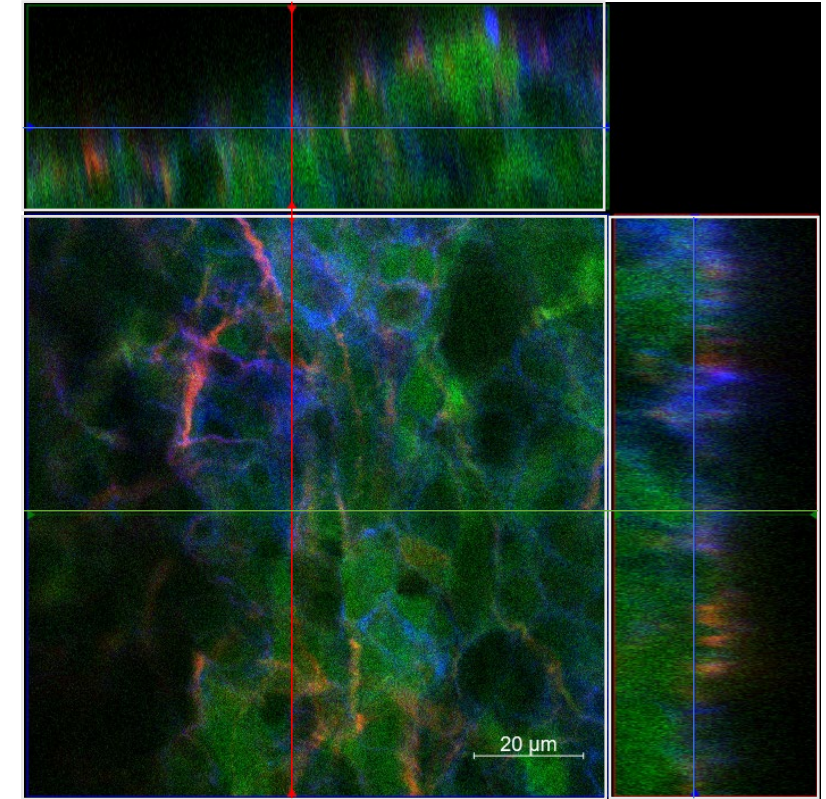

Supplement: Supplementary file 2 — Additional file 2: Figure S2. Orthogonal views of LV wholemounts. Orthogonal images of the LV wholemounts in Fig. 1 to display the localization of astrocytes and GBM cells on the LV wall surface. Scale bar = 20 μm. [file 12987_2022_354_MOESM2_ESM.pdf]

## LV-Proximal GBM

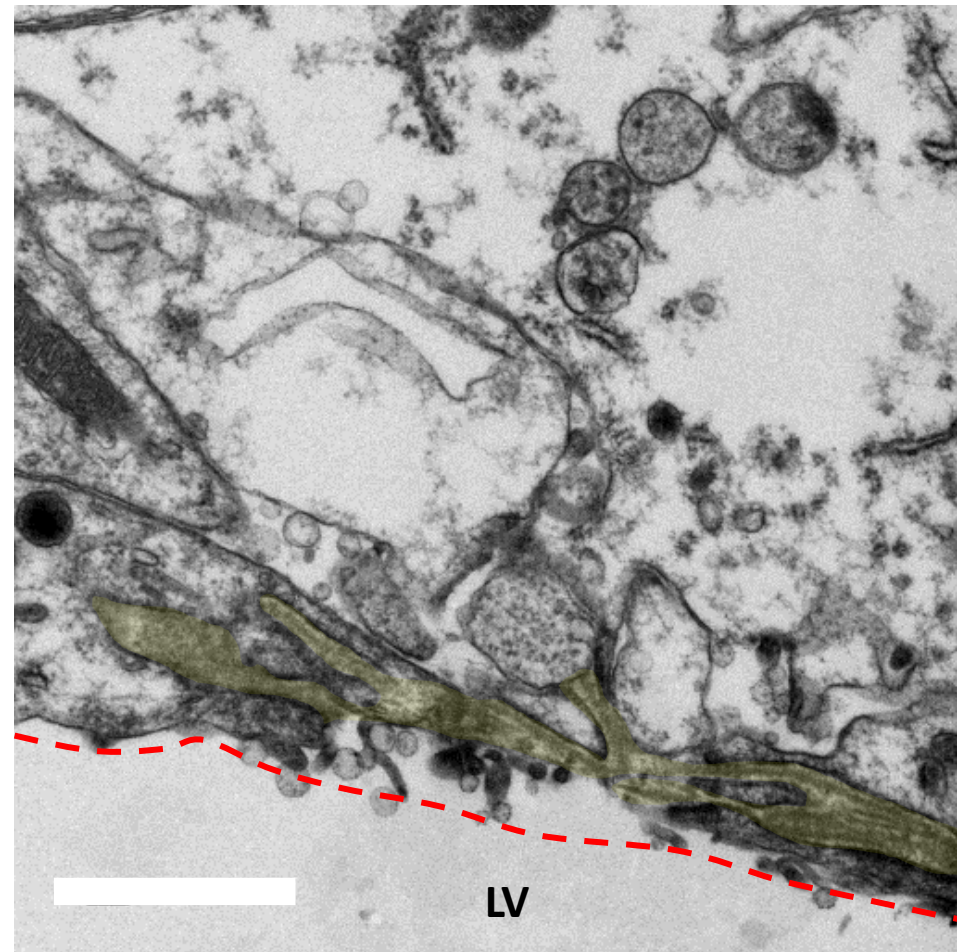

Supplement: Supplementary file 3 — Additional file 3: Figure S3. Fractones are occasionally displaced to the LV wall in the presence of LV-proximal GBM. Representative TEM image of a fractone pseudocolored in yellow in the LV-proximal GBM group displaying a fractone localized to the LV wall (outlined in red, labeled LV). Scale bar = 2 μm. [file 12987_2022_354_MOESM3_ESM.pdf]
